# Genetic testing in diffuse parenchymal lung disease

**DOI:** 10.1186/1750-1172-7-79

**Published:** 2012-10-17

**Authors:** Paolo Spagnolo, Fabrizio Luppi, Stefania Cerri, Luca Richeldi

**Affiliations:** 1 Department of Oncology, Center for Rare Lung Disease, Hematology, and Respiratory Diseases, University of Modena and Reggio Emilia, Via del Pozzo 71, Modena, 41124, Italy

**Keywords:** Genetics, Sarcoidosis, Idiopathic pulmonary fibrosis, Diffuse parenchymal lung disease

## Abstract

Diffuse parenchymal lung diseases (DPLD) represent a diverse group of disorders affecting the distal lung parenchyma, specifically the tissue and spaces surrounding the alveoli, which may be filled with inflammatory cells, proliferating fibroblasts or established fibrosis, often leading to architectural distortion and impaired gas exchange. While the underlying pathogenetic mechanisms are known or inferred for some DPLD (such as sarcoidosis, silicosis, drug reactions and collagen vascular diseases), the pathogenesis of the majority of these entities - particularly those characterized by progressive fibrosis - is poorly understood.

Several lines of evidence indicate that the development of pulmonary fibrosis is genetically determined. They include: 1. familial clustering; 2. the occurrence of pulmonary fibrosis in the context of rare inherited disorders; 3. substantial variability in the development of pulmonary fibrosis amongst individuals exposed to organic or inorganic dusts; 4. difference in susceptibility to fibrogenic stimuli amongst inbred strains of mice.

This review focuses on idiopathic pulmonary fibrosis (IPF) and sarcoidosis, the two most common DPLD and the two entities for which there is stronger evidence of a genetic predisposition, although how aberrant genes interact with each other and with environmental factors, such as smoking in IPF and infectious agents in sarcoidosis, in determining disease susceptibility and clinical phenotypes is largely unknown. Finally, we discuss practical issues and implications for both patients and physicians of recent advances in the genetics of sarcoidosis and IPF.

## Introduction

Physicians experienced in diffuse parenchymal lung diseases (DPLD) know that these disorders may run in families. DPLD are thought to be *complex* diseases, resulting from genetic variations relatively common in the general population and involving multiple genes, each contributing an effect of varying magnitude. However, an individual may have the necessary genetic profile to develop a disease and yet it will not manifest unless an environmental or infectious factor is encountered. In addition, the relative contribution of genes and environment is likely to vary in different conditions. On the other hand, the association of surfactant protein (SFTP)-C (*SFTPC*), *SFTPA2*, Telomerase Reverse Transcriptase (*TERT*), and Telomerase RNA (*TERC*) mutations with familial pulmonary fibrosis (FPF) demonstrate that a single variation may be the cause of the disease. Yet, the phenotypic heterogeneity observed among affected family members suggests that the underlying genetic abnormality(ies) may only confer a *generic* predisposition to “pulmonary fibrosis”; in such case, the phenotypic variability could be determined by the interaction of a single genetic abnormality with either different triggers (injuries/fibrogenic agents) or multiple genetic variations of smaller effects. These two pathogenetic hypotheses are not mutually exclusive.

Inheritance patterns of complex diseases are often unusual. Indeed, although many of them cluster in families, suggesting that genetics plays a major role in disease risk, not all family members are affected. Furthermore, determining a true genetic association is problematic because families share more than just their genes.

### *Complex* versus *single-gene* disorders

*Single-gene* and *complex* diseases are both characterized by multiple genetic and environmental factors. However, in single-gene disorders a specific locus has a profound effect in determining the phenotype, and may override the effects of products of other loci. Conversely, in complex diseases the phenotype results from multiple gene products combined with environmental factors. Modifying genes and genetic heterogeneity can make single-gene disorders complex but not as multifactorial as diseases that involve multiple genes and multiple environmental variables.

The main difference between single-gene and complex disorders is therefore the extent to which a single gene product disrupts homeostasis. If the gene product is so deficient or defective that it causes severe damage to the system in which it functions, the disease is generally rare and will nearly always have an early onset. Conversely, if a gene product functions adequately under most circumstances but does not when other gene products with which it is integrated fail to function, the resulting disease will be more frequent and will generally develop gradually, often presenting in middle age. Explanation of causation and determination of disease risk is more problematic with complex than with single-gene disorders. In fact, the disease risk imparted by the same gene product(s) can differ from family to family, and even amongst members of the same family, owing to heterogeneity of genes and environmental exposures (which may precipitate the disease). Table 1 summarizes the main difference between complex and single-gene disorders.

**Table 1:** Complex versus single-gene disorders

**FEATURES**	**COMPLEX**	**SINGLE GENE**
**Gene**	Segregates	Segregates
**Disorder**	Clusters	Segregates
**Number of genes/gene products**	Multiple	Primarily one (although disease modifier genes may be involved)
**Role of environmental or occupational exposures**	Important	Often overridden by effect(s) of gene mutation
**Age of onset**	Often older	Often younger
**Risk for relatives of probands**	Less predictable, often small	More predictable, often large
**Healthcare burden**	High	Relatively low
**Prevalence**	Common	Relatively rare
**Variability in expression/phenotype**	Generally more variable	Generally less variable

### Lung fibrosis. Identification of predisposing genetic factors

Fibrosis is defined by the overgrowth, hardening and/or scarring of various tissues and is attributed to excess deposition of extracellular matrix components. Fibrosis is commonly the end result of chronic inflammatory reactions induced by a variety of stimuli including persistent infections, autoimmune reactions, allergic responses, chemical insults, radiation, and tissue injury [1]. However, while this may hold true for many fibrotic DPLD, the contribution of chronic inflammation in idiopathic pulmonary fibrosis (IPF) is minimal, if any, the main argument being the failure of anti-inflammatory and immunomodulatory agents to significantly influence the course of the disease.

The existence of a genetic predisposition to lung fibrosis is suggested by a number of factors. They include: the considerable variability in developing lung fibrosis in individuals exposed to fibrogenic dusts, such as silica and asbestos, the differential response to experimentally-induced fibrosis observed in inbred mouse strains and the occurrence of pulmonary fibrosis in the context of several pleiotropic genetic disorders, such as dyskeratosis congenita, Niemann-Pick disease and Hermansky-Pudlak syndrome, amongst others [2]. However, the most persuasive evidence of a genetic signature in DPLD comes from family studies, particularly in idiopathic interstitial pneumonia (IIP) and sarcoidosis where familial aggregation has been confirmed through studies in twins, siblings raised apart, and multigenerational families [3]. The most likely mode of genetic transmission of pulmonary fibrosis in familial cases is autosomal dominant with variable penetrance [4].

### Idiopathic pulmonary fibrosis

Idiopathic pulmonary fibrosis (IPF; ORPHA2032) is the most common of the idiopathic interstitial pneumonias. The histopathologic hallmark is a heterogeneous appearance in which areas of fibrosis with scarring and honeycomb change alternate with areas of less affected or normal parenchyma (so called usual interstitial pneumonia - UIP - pattern). According to the prevailing hypothesis, IPF develops as a result of excessive, sequential lung injury and aberrant wound healing. Patients usually present with progressive dyspnoea, chronic dry cough, reticular infiltrates on chest X-ray or chest high-resolution computed tomography (HRCT) and a restrictive ventilatory defect with decreased diffusion capacity for carbon monoxide. Clinical trials of some therapeutic agents suggest a possible benefit, although to date there is insufficient evidence to support the use of any specific pharmacologic therapy for patients with IPF [5].

#### Familial pulmonary fibrosis

The occurrence of IPF in two or more members of the same family is well established, accounting for 5% of total IPF cases [6,7]. Familial and sporadic IPF are clinically and histologically indistinguishable, although familial forms may develop at an earlier age [6] and seem to have different patterns of gene transcription [8]. Cigarette smoking appears to be a risk factor for the development of the disease in FPF, suggesting that environmental/occupational exposures may accentuate genetic risk and that gene-environment interactions may be critical in IPF pathogenesis. Another remarkable finding is the pathologic heterogeneity within family members. In fact, while 50% of the families have a “uniform” diagnosis of UIP/IPF, the remaining 50% display radiological or pathologic features suggestive of a different IIP in at least one affected family member [3].

#### Surfactant protein C mutations

Mutations within surfactant protein C (*SFTPC*) have been consistently associated with FPF. Of these, the substitution of glutamine for leucine at aminoacid 188 (L188Q) in the pro-SPC protein is the most extensively studied owing to its functional relevance. In fact, while pro-SPC is normally processed through multiple steps and secreted into the alveolar space [9], L188Q mutated pro-SPC is not processed and folded correctly in the endoplasmic reticulum (ER), leading to protein accumulation and ER stress. This, in turn, activates a cascade of events known as *unfolded protein response* (UPR), a mechanism which, although designed to protect the cell, may become deleterious and lead to alveolar epithelial cell (AEC) apoptosis in case of long-standing or severe activation [10,11]. ER stress, UPR activation and AEC apoptosis have been reported in sporadic IPF, suggesting a key pathogenetic role [12]. Similarly, mutations within *SFTPA2* appear to predispose to FPF through retention of the mutant SPA form in the ER and subsequent ER stress [13,14].

#### Telomerase mutations

Telomeres - the tandem repeats of TTAGGG - represent a molecular cap of non coding DNA that protects the ends of the chromosomes against degradation. With repeated cell division, telomeres tend to shorten and chromosomes may become unstable, fused, or lost, leading to cell apoptosis. A complex of proteins and RNA called *telomerase* is essential in maintaining telomere length: the reverse transcriptase component TERT and the telomerase RNA template component TERC are key components of the *telomerase complex*[15].

Dyskeratosis congenita, a rare hereditary disorder complicated by the development of pulmonary fibrosis in 20% of patients, is associated with mutations within either *TERT*[16] or *TERC*[17] that lead to decreased telomerase activity. Recent data suggest that in a sizeable proportion of patients IPF is a disease of telomere maintenance. Mutations in *TERT* and *TERC* account for 8-15% of familial and 1-3% of sporadic cases [18,19], but short telomere length is a more common finding in IPF - with sporadic IPF cases displaying significantly shorter telomeres compared to age-matched controls - even in the absence of telomerase mutations [20]. Further, there is evidence that reduced telomere length may be a risk factor for disease outside the lung, such as liver cirrhosis or diabetes, both occurring in IPF patients at a frequency higher than expected [20,21].

#### Mucin genes

MUC5AC and MUC5B are the major gel-forming mucins found in human airway secretions. In a genome-wide linkage scan and subsequent fine mapping of a risk locus on 11p15.5, the minor allele of a common variant (rs35705950) within *MUC5B* has been found to be present in 34% of FPF cases, 38% of IPF cases and 9% of controls [22]. The rs35705950 mutant allele is associated with up-regulation of MUC5B expression in the lung, specifically in lesions of IPF, suggesting that dysregulated MUC5B expression in the lung may be involved in the pathogenesis of pulmonary fibrosis [23]. Although aberrant *MUC5B* is a plausible candidate, either by impairing mucosal host defense or interfering with alveolar repair, it is possible that unscreened genetic variants (especially in the inaccessible repetitive mucin regions) in linkage disequilibrium (LD, defined as the tendency for genetic variants located in close proximity on the same chromosome to occur together more often than expected by chance) with rs35705950 affect the function of other lung mucins.

Data from familial studies have significantly improved our understanding of IPF pathogenesis. So far, mutations in surfactant protein C, surfactant protein A2, MUC5B, telomerase reverse transcriptase and telomerase RNA component have been convincingly associated with FPF. Mutations in these genes suggest that type II AEC and cellular turn-over are central to the initiation and progression of the disease, the most plausible hypothesis being that IPF occurs due to increased cell death in the type II AEC population and/or inability of this cell population to regenerate the alveolar epithelium after injury. However, these mutations account for only 15% to 20% of FPF cases and are even less frequent in sporadic IPF. Thus, the majority of FPF cases have yet to have their genetic mutations identified and it is likely that many other genes are responsible across different families. While the data on FPF are robust, at present there are no genetic factors that have been consistently associated with sporadic IPF [24]. Microarray analysis of gene expression may ultimately contribute to elucidate disease pathogenesis and target candidates for therapy, but they are in an early phase of development [5].

### Sarcoidosis

Sarcoidosis (ORPHA797) is a systemic inflammatory disorder of unknown origin characterized histologically by tissue infiltration by mononuclear phagocytes and lymphocytes with associated non-caseating granuloma formation. According to the most convincing etiopathogenetic hypothesis, sarcoidosis occurs in genetically susceptible individuals as a consequence of exposure to one or more environmental agents. Originally described as a chronic granulomatous disorder of the skin, sarcoidosis can involve any organ, although the pulmonary manifestations typically dominate with chest radiographs being abnormal in up to 95% of patients. On the other hand, pulmonary function tests are only abnormal in a minority of patients, ranging from obstructive to restrictive pattern with or without gas exchange defects. Absolute levels of both macrophages and T-lymphocytes, the key immune effector cells in sarcoidosis, are elevated in broncho-alveolar lavage fluid (BALF) from patients, and a lymphocytosis with a CD4/CD8 ratio >3.5 is virtually diagnostic [25,26].

Familial clustering of disease was first described in two German sisters in 1923 and since then studies in various populations have identified 2.7-17% of index cases as having another affected family member [27-29]. ACCESS (A Case–Control Etiologic Study of Sarcoidosis) estimated the familial relative risk adjusted for age, gender, socio-economic status and shared environment to be 4.7 [30], confirming that family members of sarcoidosis patients have a several-fold increased risk of disease compared with the general population. In addition, monozygotic twins are more likely than dizygotic twins to have the disease, although they may exhibit only minimal concordance in terms of phenotypic features and outcomes [31]. Generally, sarcoidosis affects blacks more severely than people of other races. In addition, extra-thoracic manifestations are more prevalent in certain populations, such as chronic uveitis in U.S. blacks and Japanese, lupus pernio - a chronic rash consisting of papules and plaques usually found on the face - in Puerto Ricans, Löfgren’s syndrome in Scandinavians and myocardial involvement in Japanese. On the other hand, Löfgren’s syndrome is uncommon in blacks and Japanese [32-34].

The first reported association between sarcoidosis and specific gene products was between Class I HLA-B8 antigens and acute sarcoidosis [35]. Subsequently, HLA Class II antigens - encoded by *HLA-DRB1* and *DQB1* alleles - have been consistently associated with sarcoidosis [36-38]. However, based on the assumption that disease-associated HLA molecules present specific antigenic peptides in such a way that recognition by specific CD4^+^ T-lymphocytes results in the initiation of an abnormal inflammatory response, HLA class II genes are more likely to be involved in sarcoidosis immunopathogenesis [39]. Nevertheless, it is difficult to tease out which of the HLA Class II genes represent the primary association owing to the tight and highly variable degree of LD existing in this genomic area. Furthermore, in addition to conferring susceptibility to sarcoidosis *per se*, HLA genotypes also predispose to specific disease phenotypes, the most persuasive evidence being the association between HLA-DQB1*0201 and DRB1*0301 and Löfgren’s syndrome - defined as the acute onset of fever, erythema nodosum, bilateral hilar lymphadenopathy and polyarthralgia [40]. Conversely, results of non-HLA genes, though logical candidates based on their function, such as tumor necrosis factor (TNF)-α, chemokines and chemokine receptors, among others, have been largely inconsistent [41,42].

Candidate-gene case–control association studies have been commonly used for investigating rare diseases where recruiting large numbers of pedigrees is often difficult. The distribution of genetic variations in the genes of interest is compared between unrelated, affected individuals and matched healthy controls. This approach requires prior knowledge of a gene, function and polymorphisms, and requires the investigator to have some knowledge about the patho-physiology of the disease in question and have reason to believe that the candidate gene may influence the disease (“hypothesis-based study”). On the other hand, genome-wide association studies (GWAS) - a dense chip-based genotyping approach covering much of the human genome - permits an "agnostic" genome-wide comparison of gene-variant prevalence between cases and controls. However, because the results of GWAS are strongly influenced by the population studied, different associations have been reported in Caucasian and in African-American sarcoidosis patients, as expected [43-46].

Polymorphisms within butyrophilin-like 2 (*BTNL2*) gene - located in close proximity of the HLA complex on chromosome 6 - have been associated with sarcoidosis independently of *HLA-DRB1* alleles [47,48]. BTNL2 is thought to act as a negative co-stimulatory molecule, thus non-functional BTNL2 could theoretically result in an exaggerated T lymphocyte activation, compatible with the proposed pathophysiology of sarcoidosis [49]. However, in a number of other diseases, such as ulcerative colitis, multiple sclerosis, type 1 diabetes, rheumatoid arthritis, systemic lupus erythematosus, Graves’ disease, tuberculosis, leprosy, Crohn’s disease as well as in a study of sarcoidosis, the *BTNL2* association appears to be “driven” by various *HLA-DRB1* alleles in LD with the non-functional rs2076530 A allele [50-54], highlighting the difficulty in identifying the precise risk locus/i when HLA genes and nearby loci in LD are considered simultaneously.

### Disorders in which genetic testing is highly recommended

#### Hermansky-Pudlak syndrome

Hermansky-Pudlak syndrome (HPS; ORPHA79430) is a rare autosomal recessive disorder characterized by oculo-cutaneous albinism, bleeding diathesis resulting from platelet storage pool deficiency, and lung fibrosis [55]. The disease, which is caused by defects of multiple cytoplasmic organelles, including melanosomes, platelet-dense granules and lysosomes, has a prevalence of 1 in 1,800 in Puerto Rico with only isolated case reports and small case series having been reported in the rest of the world [56]. A history of bruising, heavy menstrual cycles and fair skin should raise the suspicion of HPS. Once the diagnosis has been confirmed - by demonstration of the absence of platelet dense bodies on whole-mount electron microscopy - other family members should be screened for the syndrome and mutations within genes known to cause the disease (*HPS1-HPS8*) searched for [57].

#### Lymphangioleiomyomatosis and Tuberous Sclerosis Complex

Lymphangioleiomyomatosis (LAM; ORPHA538) is a rare cystic lung disease characterized by an aberrant proliferation of smooth muscle-like cells (“LAM cells”), and associated with renal angiomyolipomas and lymphatic spread [58]. LAM can occur either as an isolated disorder or in 1-3% of patients with tuberous sclerosis complex (TSC; ORPHA805), an autosomal dominant systemic disorder resulting from mutations in the *TSC1* and *TSC2* gene and characterized by epilepsy, widespread hamartomatous lesions, renal angiomyolipomas, skin lesions and mental retardation [59]. Both isolated LAM and LAM in the context of TSC have a remarkable female gender restriction with patients being affected generally in their reproductive age, while TSC is equally distributed amongst genders. Lung manifestations - in the form of profuse round, thin-walled cysts - are indistinguishable in isolated LAM and in LAM in the context of TSC [60]. Mutations in *TSC1* are found in 15-30% of familial cases and 10-15% of sporadic cases, while mutations in *TSC2* occur more frequently, accounting for 75-80% of all sporadic cases [61,62]. Once the diagnosis of LAM has been confirmed - by the demonstration of the immunoreactivity of LAM cells with HMB45 antibody - mutations in *TSC1* and *TSC2* genes should be searched for. In fact, in patients with LAM raising in the context of TSC careful screening of family members may reveal other affected - often asymptomatic - individuals.

#### Birt-Hogg-Dubé syndrome

Birt-Hogg-Dubé (BHD; ORPHA122) syndrome is an autosomal dominant disorder caused by loss-of-function mutations in the folliculin (*FLCN*) gene and characterized by skin fibro-folliculomas, multiple lung cysts lined by fibrous band (in up to 90% of cases), spontaneous pneumothorax, and renal cancer [63]. BHD syndrome-associated skin lesions include angiofibromas, which are more typically associated with tuberous sclerosis. In turn, tuberous sclerosis may manifest with pneumothorax (caused by rupture of lung cysts), and renal angiomyolipomas and should therefore be considered in the differential diagnosis of BHD syndrome [64]. Because the skin lesions usually precede renal malignancies by several years, a correct diagnosis may allow early diagnosis of BHD syndrome and screening for renal cancer in other family members.

#### Dyskeratosis congenita

Dyskeratosis congenita (DC; ORPHA1775) is a rare systemic disease usually presenting in the first or second decade with bone marrow failure and a triad of muco-cutaneous lesions including abnormal pigmentation, dystrophic nails, and oral leukoplakia [65]. DC is considered a syndrome of premature aging as suggested by other common features, such as premature graying of the hair, pulmonary fibrosis (which develops in 20% of cases), testicular atrophy, cryptogenic cirrhosis, osteoporosis, and increased risk of malignancy. In favor of this hypothesis, DC was the first disease recognized to result from impaired telomere maintenance [66]. Mode of inheritance may be autosomal dominant, recessive, or X-linked; the X-linked form results from a mutation in the *DKC1* gene, which encodes dyskerin, a telomerase-associated protein. Although an identifiable mutation is present in roughly 40% of cases, telomere length is uniformly reduced in patients with DC. In addition, mutations within *TERT* and *TERC* in DC kindreds have been associated with *genetic anticipation* - the occurrence of more severe and earlier onset disease in later generations secondary to progressive telomere shortening [17].

Pulmonary involvement from rare diseases represents one end of a spectrum of clinical manifestations. Making the correct diagnosis is crucial for both preventing fatal complications in affected subjects and early diagnosing the disease in other family members.

### What is the utility of genetic testing in sarcoidosis and IPF?

The utility of genetic testing stems on the possibility to predict disease development in susceptible individuals. However, the risk-to-benefit ratio for genetic screening is related to its pre-test probability and should carefully be evaluated for each disorder. As such, genetic testing is strongly recommended, for instance, in BHD syndrome because there is no locus heterogeneity (only one culprit gene is known) and the presence of a mutation advocates screening for renal cancer, which complicates 10% of cases [67]. Conversely, routine genetic testing is of unproven benefit in sporadic LAM and is not recommended by current guidelines [68].

In highly specialized referral centres for sarcoidosis, patients presenting with Löfgren’s syndrome are routinely genotyped for the HLA-DRB1*0301/DQB1*0201 (DR3) haplotype. In fact, in addition to displaying typical clinical features - acute onset, bilateral hilar lymphadenopathy, erythema nodosum and/or bilateral ankle arthritis - this subset of patients can be further characterized according to the carriage of DR3 as disease resolution occurs in almost every DR3-positive patient but only in half of those who do not carry this haplotype [69].

Testing for rare and common variants is different: mutations within *TERT*, *TERC*, *SFTPC* and *SFTPA2* are individually very rare, but have a large effect in the kindreds in which they are found. In fact, inheritance of these mutations segregate with pulmonary fibrosis in a Mendelian autosomal dominant pattern with reduced penetrance [70]. Conversely, the minor allele of the *MUC5* rs35705950 polymorphism is present in 9% of the normal population. As such, testing for the *MUC5* rs35705950 mutant allele could be valuable only in selected individuals with high pre-test risk of developing the disease (i.e., members of families subject to idiopathic interstitial pneumonia), as the positive predictive value of such testing would be very low.

Results of genetic testing are often complex and difficult to interpret in isolation. Genetic counselling is essential in providing a personalized interpretation of the results, with special emphasis on the meaning of *susceptibility*, the risk for other family members to develop the disease and the limited predictive value of positive and negative results. Once provided with this information, an individual can make more informed decisions regarding their healthcare. In addition, those found to be at increased risk should utterly avoid any (further) potentially harmful exposures.

Should a respiratory physician consider routine genetic testing in sarcoidosis and IPF? At present, susceptibility testing for these diseases is neither widely available nor recommended by current guidelines in patients with familial or sporadic disease as part of their clinical evaluation. Similarly, there is no evidence that unaffected family members should be screened for asymptomatic disease. This is particularly true for dominant mutations of variable penetrance where prediction of risk is problematic. Furthermore, it is unclear whether early diagnosis makes the disease more amenable to therapeutic intervention [5]. Considering the average costs of genetic test and the possibility of identifying variants of unknown significance, genetic screening should be limited to selected disorders, i.e., those displaying no or low genetic locus heterogeneity and for which a specific diagnosis would impact patient management.

### Future directions

The recent development of next generation sequencing technologies (whole genome, whole exome and targeted region sequencing) is likely to rapidly increase the number of genetic variants associated with sarcoidosis and IPF, including rare risk alleles, which cannot be identified by genotyping. In fact, a significant amount of heritability in sarcoidosis and IPF could be accounted for by rare variants each with moderate to high penetrance [71]. Genetic data should be integrated with gene expression and epigenetic data in order to prioritize candidates for further studies. Once prioritized, candidate genes will then need to be evaluated for their role in disease pathogenesis (i.e., in cellular and animal studies). If genetic links with a disease are clearly defined, then the pathways involved with these genes and gene products could be specifically targeted with potentially tremendous therapeutic implications. The success of sirolimus - a mammalian target of rapamycin (mTOR) signaling inhibitor - in stabilizing lung function, reducing respiratory symptoms and improving quality of life in tuberous sclerosis/LAM patients is proof of concept that therapy targeting specific defective genetic and biochemical pathways can be successful [72]. The hope is that treatment strategies targeting individual aberrant genes will be soon available for other DPLD with genetic signature.

We have incomplete knowledge of ethnic differences in genetic associations as well as limited insight in gene-gene and gene-environment interactions. Future studies should address these deficiencies. To this end, participation in international consortia of institutions - with the intention of recruiting larger numbers of patients - is critical. Likewise, it is imperative that meticulous databases of phenotypically well-defined patients are continued to be constructed as this will significantly reduce the number of subjects required to show meaningful genetic associations. In fact, relatively small studies based on accurate genotyping with exhaustively defined phenotype criteria are equally, if not more so, able to detect the same effect as larger studies of a less stringent design. It is possible that genetics extends to determining not only overall susceptibility but also distinct phenotypic routes, and that genes responsible for the development of a given disease are different from those determining its phenotypic expression. As such, it is essential that genetic data are always analyzed according to clinical phenotype and not limited to a “generic” disease susceptibility. Last, but not least, data regarding incidence and prevalence of familial cases of pulmonary fibrosis and sarcoidosis are incomplete, at best, as the disease may be asymptomatic, thus undiagnosed. Future studies should also address and clarify this issue.

## Abbreviations

ACCESS: A Case–control Etiologic Study of Sarcoidosis; AEC: Alveolar Epithelial Cell; BALF: Broncho-Alveolar Lavage Fluid; BHD: Birt-Hogg-Dubé; BTNL2: Butyrophilin-like 2; DPLD: Diffuse Parenchymal Lung Disease; DC: Dyskeratosis Congenita; ER: Endoplasmic Reticulum; FLCN: Folliculin; FPF: Familial Pulmonary Fibrosis; GWAS: Genome-Wide Association Study; HPS: Hermansky-Pudlak Syndrome; HRCT: High-Resolution Computed Tomography; IIP: Idiopathic Interstitial Pneumonias; IPF: Idiopathic Pulmonary Fibrosis; LD: Linkage Disequilibrium; LAM: Lymphangioleiomyomatosis; mTOR: Mammalian Target of Rapamycin; SFTP: Surfactant Protein; TERC: Telomerase RNA; TERT: Telomerase Reverse Transcriptase; TNF: Tumor Necrosis Factor; TSC: Tuberous Sclerosis Complex; UIP: Usual Interstitial Pneumonia; UPR: Unfolded Protein Response.

## Competing interests

The authors declare that they have no competing interests.

## Authors’ contributions

PS conceived of the study and drafted the manuscript. FL, SC and LR participated in the design of the review and helped to draft the manuscript. All authors read and approved the final manuscript.
